# Effect of abrupt weaning at housing on leukocyte distribution, functional activity of neutrophils, and acute phase protein response of beef calves

**DOI:** 10.1186/1746-6148-6-39

**Published:** 2010-07-22

**Authors:** EM Lynch, B Earley, M McGee, S Doyle

**Affiliations:** 1 Animal and Bioscience Research Department, Animal & Grassland Research and Innovation Centre, Teagasc, Grange, Dunsany, Co Meath, Ireland; 2 Department of Biology and National Institute for Cellular Biotechnology, National University of Ireland Maynooth, Co. Kildare, Ireland; 3 Livestock Systems Research Department, Animal & Grassland Research and Innovation Centre, Teagasc, Grange, Dunsany, Co Meath, Ireland

## Abstract

**Background:**

Sixteen, spring-born, single suckled, castrated male calves of Limousin × Holstein-Friesian and Simmental × Holstein-Friesian dams respectively, were used to investigate the effect of weaning on total leukocyte and differential counts, neutrophil functional activity, lymphocyte immunophenotypes, and acute phase protein response. Calves grazed with their dams until the end of the grazing season when they were housed in a slatted floor shed. On the day of housing, calves were assigned to a treatment, (i) abruptly weaned (W: *n *= 8) or (ii) non-weaned (controls) (C: *n *= 8). Weaned calves were housed in pens without their dams, whereas non-weaned (control) calves were housed with their dams. Blood was collected on day -7, 0 (housing), 2, 7, and 14 to determine total leukocyte and differential counts and concentration of fibrinogen and haptoglobin. Lymphocyte immunophenotypes were characterised using selected surface antigens (CD4^+^, CD8^+^, WC1^+ ^(γδ T cells), MHC Class II^+ ^lymphocytes), and the functional activities of neutrophils (surface expression of L-selectin (CD62L), phagocytic and oxidative burst activity) were investigated using flow cytometry.

**Results:**

Treatment × sampling time interactions (*P *< 0.05) were detected for total leukocyte and neutrophil counts, all lymphocyte subsets, mean fluorescence intensity of CD62L^+ ^neutrophils, and percentage neutrophils performing phagocytosis. On d 2, total leukocyte and neutrophil count increased (*P *< 0.001), and percentage CD4^+ ^and CD8^+ ^lymphocytes, percentage phagocytic neutrophils, mean fluorescence intensity of CD62L^+ ^neutrophils decreased (*P *< 0.05) in W compared with baseline (d 0), whereas they were unchanged (*P *> 0.05) in C. On d 2, percentage WC1^+ ^lymphocytes decreased (*P *< 0.05), whereas percentage MHC class II^+ ^lymphocytes increased (*P *< 0.05) in W and C, however the magnitude of change was greater in W than C. There were no treatment × sampling time interactions (*P *> 0.05) for monocyte, eosinophil, and basophil counts, percentage G1^+ ^neutrophils, or percentage oxidative burst positive neutrophils.

**Conclusions:**

Abrupt weaning resulted in increased neutrophil counts and impaired trafficking and phagocytic function. Together with the changes in lymphocyte subsets, the results suggest that there was a greater transitory reduction in immune function at housing in abruptly weaned than non-weaned beef calves.

## Background

Weaning is an inherent husbandry practice in cow-calf beef production systems that imposes physical, psychological, and nutritional stressors on calves. Integrated calf-to-beef production systems, such as seasonal grass-based systems, often combine weaning and housing [1], whereas non-integrated systems often combine weaning with additional stressors such as transportation and marketing, prior to entry into feedlots [2]. Following abrupt weaning, beef calves exhibit distress behaviours [3,4], with alterations in hormonal mediators of stress [5,6] and immune function [7-9] evident up to 7 d post-weaning. Furthermore, weaning is considered to be a predisposing factor to bovine respiratory disease (BRD) [10,11].

Neutrophils provide the first line of cellular defence against pathogens, whereas lymphocytes are of pivotal importance in cell-mediated and humoral immunity [12,13]. Although studies have examined neutrophil and lymphocyte function and distribution following transport [14,15], and during natural and experimental cases of BRD [16-18], none have investigated the direct effects of weaning on these immune variables in beef calves. Additional information on the immune status of newly weaned calves at a time when pathogen exposure is heightened may be useful for identifying animals likely to succumb to infection.

Thus, the objectives of the study were to examine the effect of abrupt weaning at housing on i) peripheral leukocyte and differential counts, ii) granulocyte positive neutrophils and lymphocyte immunophenotypes, iii) phagocytic and oxidative burst activity, and surface expression of CD62L of neutrophils, and iv) the acute phase protein response in beef calves.

## Results

### Rectal body temperature

There was no effect of treatment (*P *= 0.4) or treatment × sampling time interaction (*P *= 0.3), for rectal body temperature whereas sampling time was significant (*P *= 0.048) (data not shown). Rectal body temperature increased (*P = *0.02) on d 2 (mean (s.e.) 38.9 (0.09) °C), and subsequently did not differ (*P *> 0.05) compared with baseline (d 0; mean (s.e) 38.5 (0.07) °C).

### Total leukocyte and differential counts

There was a treatment × sampling time interaction (*P = *0.01) for total leukocyte count whereby on d 2 it increased (*P *= 0.004) in W and returned to baseline, whereas C did not differ (*P *= 0.9) from baseline (Table 1). There were treatment × sampling time interactions for neutrophil (*P <*0.0001) and lymphocyte (*P *= 0.002) counts (Table 1). On d 2, neutrophil count increased (*P *< 0.0001) and lymphocyte counts decreased (*P *= 0.008) in W, whereas C did not differ from baseline (Table 1). There were no effects (*P >*0.05) of treatment and sampling time, or treatment × sampling time interactions for monocyte, eosinophil and basophil counts (Table 1).

**Table 1:** Effect of abrupt weaning at housing on total leukocyte and differential counts in beef calves.

		Day (d) relative to housing					*P*-values^1^		
		
Cell type (× 10^3^cells/μL)		-7	0^2^	2	7	14	T	S	T × S
Total leukocytes	C	10.3 ± 0.61	10.5 ± 0.31	10.8^x ^± 0.47	10.5 ± 0.92	10.3 ± 0.32	0.03	0.0005	0.01
	W	10.6 ± 0.62	10.3^a ^± 0.31	13.3^b, c, y ^± 0.47	11.2^a, c ^± 0.92	10.2^a ^± 0.32			
Neutrophils	C	2.8 ± 0.11	2.9 ± 0.17	3.2^x^ ± 0.65	2.9 ± 0.64	2.5 ± 0.23	0.006	< 0.0001	< 0.0001
	W	2.7 ± 0.11	2.5^a ^± 0.18	6.6^b, y ^± 0.68	2.7^a ^± 0.64	2.7^a ^± 0.24			
Lymphocytes	C	7.0 ± 0.19	6.7 ± 0.21	6.6^x ^± 0.22	6.8 ± 0.49	6.9 ± 0.36	0.3	0.0006	0.002
	W	6.9 ± 0.19	6.9^a ^± 0.22	5.2^b, y ^± 0.22	7.3^a ^± 0.49	6.7^a ^± 0.37			
Monocytes	C	0.64 ± 0.10	0.46 ± 0.05	0.59 ± 0.07	0.54 ± 0.11	0.47 ± 0.05	0.5	0.1	0.16
	W	0.60 ± 0.11	0.48 ± 0.06	0.59 ± 0.06	0.59 ± 0.10	0.50 ± 0.05			
Eosinophils	C	0.29 ± 0.05	0.33 ± 0.10	0.31 ± 0.12	0.59 ± 0.11	0.35 ± 0.07	0.3	0.06	0.4
	W	0.21 ± 0.05	0.31 ± 0.11	0.38 ± 0.13	0.29 ± 0.10	0.24 ± 0.07			
Basophils	C	0.12 ± 0.02	0.15 ± 0.02	0.13 ± 0.01	0.11 ± 0.02	0.12 ± 0.01	0.6	0.5	0.8
	W	0.11 ± 0.02	0.12 ± 0.02	0.12 ± 0.01	0.11 ± 0.02	0.13 ± 0.01			

^1^T = treatment, S = sampling time, T × S = treatment × sampling time interactions.

W = abruptly weaned, C = non-weaned (control) calves.

^a, b, c^Within a row, least squares means without a common superscript differ (*P <*0.05).

^x, y^Within a column (day) for each variable, least squares means without a common superscript differ (*P <*0.05). ^2^Baseline is defined as d 0 for each variable. Data presented are least squares means ± s.e.m.

### Granulocyte-positive cells

There was no effect of treatment (*P *= 0.1) or treatment × sampling time interaction (*P *= 0.2) for percentage of G1^+ ^neutrophils but sampling time was significant (*P *< 0.0001). On d 2, the percentage of G1^+ ^neutrophils it increased (*P *< 0.001, mean (s.e.) 48.5 (1.71) MFI)) and subsequently returned to baseline (mean (s.e) 37.3 (1.7) MFI) by d 7 (Table 2).

**Table 2:** Effect of abrupt weaning at housing on percentage G1^+ ^neutrophils and CD62L^+ ^neutrophils MFI^1 ^in beef calves.

		Day (d) relative to housing					*P*-values^2^		
		
Neutrophils		-7	0^2^	2	7	14	T	S	T × S
G1^+^, %	C	27.6 ± 0.68	27.3^a ^± 0.83	35.0^b, c, x ^± 0.77	30.8^a, c ^± 0.84	27.7^a ^± 0.85	0.1	< 0.0001	0.2
	W	28.9 ± 0.68	27.4^a ^± 0.83	41.9^b, y ^± 0.77	31.4^a ^± 0.84	28.2^a ^± 0.85			
CD62L^+^, MFI^1^	C	120.9 ± 1.81	121.1 ± 1.91	119.8^x ^± 2.31	122.3 ± 2.02	120.4 ± 1.49	0.1	< 0.0001	0.002
	W	122.6 ± 1.81	122.2^a ^± 1.91	107.0^b, y ^± 2.31	119.3^a ^± 2.02	121.3^a ^± 1.49			

^1^MFI = mean fluorescence intensity

^2^T = treatment, S = sampling time, T × S = treatment × sampling time interactions.

W = abruptly weaned, C = non-weaned (control) calves.

^a, b, c^Within a row, least squares means without a common superscript differ (*P <*0.05).

^x, y^Within a column (day) for each variable, least squares means without a common superscript differ (*P <*0.01). ^2^Baseline is defined as d 0 for each variable. Data presented are least squares means ± s.e.m.

### Neutrophil functional activity

There was a treatment × sampling time interaction (*P = *0.002) for MFI of CD62L^+ ^neutrophils (Table 2). On d 2, MFI decreased (*P *= 0.002) in W and subsequently returned to baseline, whereas C did not differ (*P = *0.9) from baseline. There was a treatment × sampling time interaction (*P *= 0.02) for percentage phagocytosing neutrophils (Table 3). On d 2, percentage neutrophils performing phagocytosis decreased in W (*P *< 0.01), whereas C did not differ (*P *= 0.4) compared with baseline and subsequently, they returned to baseline (*P *> 0.05). There was no effect of treatment (*P *= 0.2) and sampling time (*P *= 0.5), or treatment × sampling time interaction (*P *= 0.7) for the percentage neutrophils positive for oxidative burst activity (Table 3).

**Table 3:** Effect of abruptly weaning at housing on phagocytic and oxidative burst positive neutrophils in beef calves.

		Days (d) relative to housing					*P*-values^1^		
		
Neutrophils, %		-7	0^2^	2	7	14	T	S	T × S
Phagocytosis positive	C	87.2 ± 2.00	88.8 ± 1.98	79.4^x ^± 3.20	87.4 ± 2.48	89.6 ± 2.18	0.003	< 0.0001	0.02
	W	87.0 ± 2.00	89.6^a ^± 1.98	61.3^b, y ^± 3.20	79.7^a ^± 2.48	89.3^a ^± 2.18			
Oxidative burst positive	C	36.5 ± 3.93	36.6 ± 2.14	45.1 ± 4.83	46.1 ± 5.24	40.3 ± 5.82	0.5	0.2	0.7
	W	34.7 ± 3.93	37.0 ± 2.14	41.3 ± 4.83	37.2 ± 5.24	40.1 ± 5.82			

^1^T = treatment, S = sampling time, T × S = treatment × sampling time interactions.

W = abruptly weaned, C = non-weaned (control) calves.

^a, b, c^Within a row, least squares means without a common superscript differ (*P = *0.01).

^x, y^Within a column (day) for each variable, least squares means without a common superscript differ (*P = *0.03). ^2^Baseline is defined as d 0 for each variable. Data presented are least squares means ± s.e.m.

### Lymphocyte immunophenotypes

**Figure 1:**
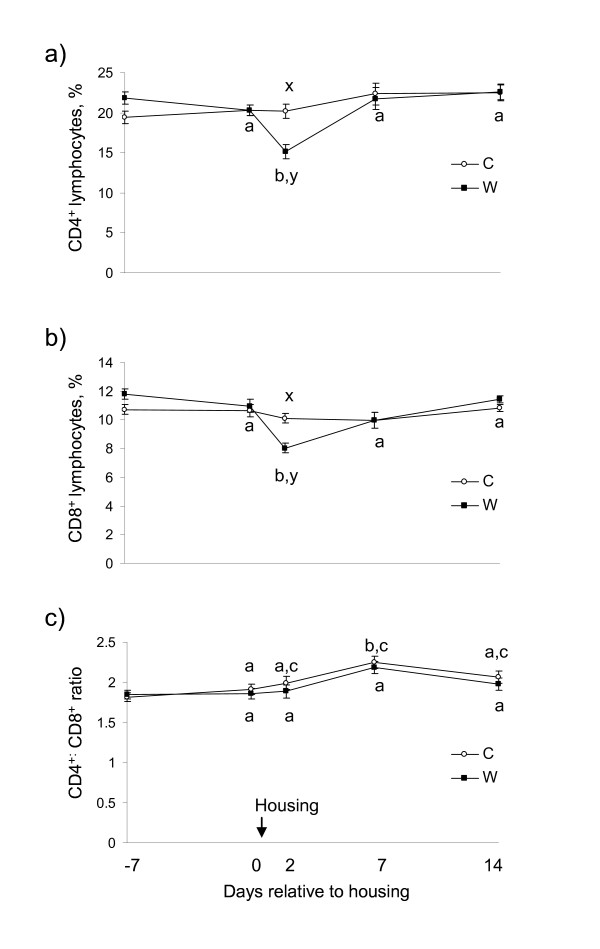
**Effect of abrupt weaning at housing on percentage a) CD4^+ ^and b) CD8^+ ^lymphocytes (least squares mean ± s.e.m., %) and c) CD4: CD8 ratio (least squares means ± SEM) in beef calves**. W = abruptly weaned calves, C = non-weaned (control) calves. ^a, b, c^Between days, least squares means without a common superscript differ (*P <*0.05). ^x, y^Within a day, least squares means without a common superscript differ (*P <*0.05). T × S = treatment × sampling time point interaction. Baseline is defined as d 0 for each variable.

There was a treatment × sampling time interaction for percentage CD4^+ ^(*P = *0.0002) and CD8^+ ^(*P *< 0.0001) lymphocytes. On d 2, percentage CD4^+ ^and CD8^+ ^lymphocytes decreased (*P *< 0.001) in W and subsequently returned to baseline (*P *> 0.05), whereas C did not differ (*P *> 0.05) from baseline (Figure 1a and 1b). There was no effect of treatment (*P *= 0.3) or treatment × sampling time interaction (*P *= 0.6) for CD4: CD8 ratio. Sampling time was significant (*P *= 0.005), whereby CD4: CD8 ratio increased (*P = *0.05) on d 7 compared with baseline (Figure 1c).

**Figure 2:**
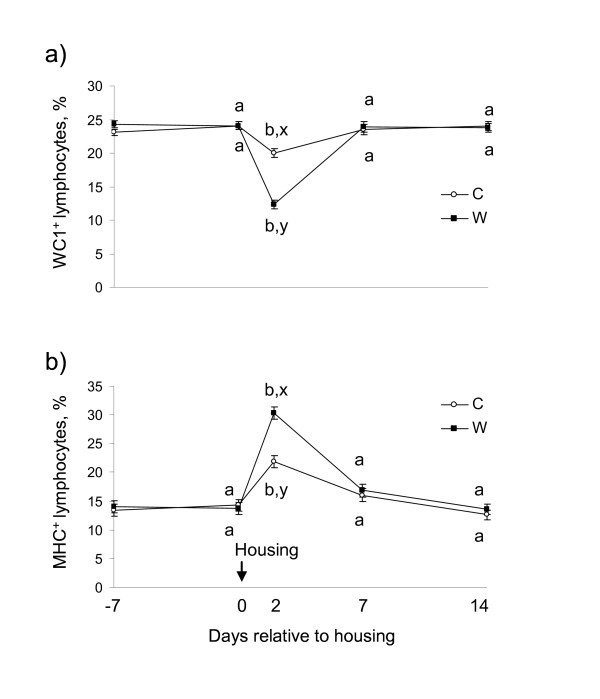
**Effect of abrupt weaning at housing on a) WC1^+ ^and b) MHC Class II^+ ^lymphocytes (least squares mean ± s.e.m., %) in beef calves**. W = abruptly weaned calves, C = non-weaned (control) calves. ^a, b, c^Between days, least squares means without a common superscript differ (*P <*0.001). ^x, y^Within a day, least squares means without a common superscript differ (*P <*0.01). T × S = treatment × sampling time point interaction. Baseline is defined as d 0 for each variable.

There was a treatment × sampling time interaction for WC1^+ ^(*P <*0.001) and MHC Class II^+ ^(*P *= 0.002) lymphocytes. On d 2, the percentage WC1^+ ^lymphocytes decreased (*P <*0.005) in W and C, however the decrease was greater (*P *= 0.008) in W (Figure 2a). Subsequently, the percentage WC1^+ ^lymphocytes returned to baseline in both treatments (*P >*0.05). Conversely, on d 2, percentage MHC Class II^+ ^lymphocytes increased (*P <*0.01) in W and C but the increase was greater (*P *= 0.002) in W than C, before returning to baseline (*P *= 0.3) (Figure 2b).

### Acute phase proteins

There were no effects (*P *> 0.05) of treatment or treatment × sampling time interactions for plasma fibrinogen and haptoglobin concentrations. Sampling time was significant (*P *= 0.0002) for haptoglobin, whereby on d 2, concentration increased (*P *= 0.01) compared with baseline (means (s.e.) 0.48 (0.033) mg/mL versus 0.32 (0.023) mg/mL). Sampling time was not significant (*P *= 0.3) for fibrinogen concentration (data not shown).

## Discussion

The immune variables measured in the present study showed that weaning together with movement of beef calves from a pasture environment to a housing environment elicited transient neutrophilia, impaired neutrophil phagocytic function, decreased peripheral lymphocyte count and altered immunophenotypes. To the authors' knowledge, this is the first study to collectively examine these immune responses to weaning and housing in the beef calf.

Stress-induced changes in immune function have been documented in cattle, with alterations to cell-mediated and humoral immunity having a significant impact on immunocompetence which may render an animal more susceptible to infection [19-22]. In the present study, neutrophil surveillance and phagocytic activity were affected by weaning, however bactericidal activity was unaffected. Post-weaning, peripheral neutrophil count increased which was most likely resultant of the decreased surface expression of L-selectin as defined by the reduced MFI CD62L on blood neutrophils. Reduction of this adhesion molecule from the surface of neutrophils, where it is constitutively expressed on resting cells prior to activation, suggests that weaning may have had a negative impact on the ability of these cells to roll along and adhere to the endothelium lining of blood vessels and thus, more cells remained in circulation as evidenced by neutrophilia. This phenomenon has been documented in physiologically stressed cattle, most notably coinciding with parturition [23-25] and following transportation [26].

Additionally, weaned calves had reduced percentage of neutrophils performing phagocytosis compared with control calves. This transient depression in phagocytosis was short lived with cells recovering this function by 7 d post-housing. This finding concurs with the other findings that report a transient decrease in neutrophil phagocytic activity in weaned foals [27]. Decreased phagocytosis has also been reported in horses following transportation [28], in cows that were milked only once-a-day [29], and in dairy cattle following administration of ACTH and dexamethasone [30-32]. Although phagocytic activity of neutrophils was impaired immediately post-weaning, the ability of these cells to perform oxidative burst activity was unaffected, implying that any internalised particles or bacteria could be efficiently degraded despite the depression in particle uptake by neutrophils. Unaltered respiratory burst activity was reported in 14 mo old Holstein-Friesian bulls that were subjected to either Burdizzo castration or hydrocortisone infusion [33]. Moreover, *in vitro *studies examining pharmacological high doses of infused hydrocortisone and therapeutic doses of glucocorticoids did not affect the oxidative burst activity of neutrophils isolated from high milk yielding Holsteins 2 - 5 weeks post-partum [34].

Appropriate lymphocyte responses provide immunoprotection against bacterial and viral antigens but can also critically influence immunopathogenesis of disease [35-37]. In agreement with other studies [6,7,38], weaning decreased peripheral lymphocyte count. Further investigation of the lymphocyte immunophenotypes showed that there was a greater reduction in the percentage of γδ T cells than αβ T cells post-weaning. Transient decreases in proportion of WC1^+ ^lymphocytes were observed in both treatment groups, with the greatest degree of change observed when weaning and housing were combined. Calves held in conventional housing had significantly lower percentage WC1^+ ^T cells compared with calves that were allowed free-range grazing and access to their dams [39]. Moreover, a decrease in WC1^+ ^T cells in the free-ranging calves coincided with a change from an outdoor to indoor environment following a period of transportation. Contrary to the present findings, percentage WC1^+ ^T cells did not return to baseline in that study, rather they remained decreased and after 3 weeks post-housing were not different from calves that were permanently housed [39]. The additional stressor of transportation may have contributed to the prolonged response observed in the latter study. Our findings are in agreement with other studies which have shown that γδ T cells are most sensitive to physiological and pharmacological stressors with reductions in percentage WC1^+ ^lymphocytes observed following transportation of beef steers [14], parturition in dairy cows [40] and dexamethasone treatment in dairy and beef bulls [41,42].

In the present study, the decrease in percentage αβ T cells post-weaning was evidenced by transient decreases in percentage CD4^+ ^and CD8^+ ^lymphocytes. Decreases in both peripheral CD4^+ ^and CD8^+ ^lymphocytes have been reported in calves at unloading after 14 h truck transportation, following which they returned to pre-transport values by 24 h [14], and in beef steers following induction into a feedlot environment [43]. Some studies examining the effect of stress in domestic animals have utilised the CD4^+^: CD8^+ ^ratio as an indicator of stress-induced immunosuppression, however conflicting evidence exists in cattle [44-47]. Our findings showed that movement of beef calves from a pasture environment to a housing environment was able to elicit a transient increase in CD4^+^:CD8^+ ^ratio. This increase may reflect recovery of immune competence in an attempt to restore homeostasis following the initial reaction to the onset of stress. An overall rise in CD4^+^: CD8^+ ^ratio was reported in sheep that were housed either in isolation or in groups [47]. These authors attributed the rise in CD4^+^:CD8^+ ^ratio to recovery of immune compensation following perturbation to lymphocyte subsets due to a stressful change in the environment. Thus, CD4^+^:CD8^+ ^ratio may provide more information on the recovery of homeostasis rather than occurrence of immunosuppression. Further research into the utility of this ratio to describe a stress scenario in domestic animals is warranted.

In the present study, percentage MHC class II^+ ^lymphocytes in peripheral blood increased significantly in weaned calves and to a lesser extent in control calves indicating that a change in environment in combination with and without weaning resulted in the activation of lymphocytes. This increase in percentage MHC class II^+ ^lymphocytes may be due to increased B cells or activated T cells in circulation [48]. Increase in MHC class II^+ ^lymphocytes may be due to expansion of NK cells bearing MHC class II molecules [49], however, this supposition cannot be confirmed by the findings of the present study as NK cells were not investigated. Our findings concur with Riondato et al. (2008) [14] who reported increased percentage MHC class II^+ ^cells in transported beef steers, but in that study the response was more prolonged (> 7 d). Holstein bulls challenged with pharmacological doses of dexamethasone were reported to have increased percentage MHC class II^+ ^cells compared with non-treated controls [50]. Furthermore, increased percentage MHC class II^+ ^lymphocytes have been reported following repeated restraint and isolation stress in lambs [51].

The reduction in lymphocyte subsets is most likely attributable to a redistribution of these cells from the peripheral circulation to immune compartments or tissues of greater importance during a stressful event. Trafficking of cells is an important and dynamic factor for effective cell-mediated immunity and stress has been shown to influence this process [52-54].

It is important to note that despite the numerous changes in leukocyte counts, neutrophil function and lymphocyte immunophenotypes observed, generally these perturbations were transient and homeostasis was restored by 7 d post-weaning. Additionally, changes in some of the immune-related biomarkers, namely the proportion of γδ T cells and MHC class II^+ ^cells in peripheral circulation and the phagocytic function of blood neutrophils, were evident following change in environment alone. Further research is warranted to investigate the potential use of these measures as prognostic biomarkers of stress sensitive and consequently, infection susceptible animals in response to other stressors.

In line with the previous study, total leukocyte count increased following weaning [6]. Monocyte, basophil and eosinophil counts were not sensitive to weaning or change in environment as evidenced by the unaltered profiles pre- and post-weaning.

Previous studies have shown that weaning induces an acute phase protein response in beef calves [9,55,56], whereas in the present study, although there was a numerical increase in fibrinogen and haptoglobin concentration, it was not statistically significant. Increased and unchanged concentration of fibrinogen and haptoglobin, respectively in weaned bull calves has been reported [7], whereas elevated responses in weaned steers have been reported for fibrinogen [6,55] and haptoglobin [8,9,55].

## Conclusions

Abrupt weaning at housing affected total leukocyte, neutrophil and lymphocyte counts, lymphocyte immunophenotypes and functional activity of neutrophils in beef calves. Neutrophilia was evident post-weaning, however, crucially, the functional capacity of these cells to effectively traffic from the periphery and phagocytose bacteria was temporarily decreased. Additionally, weaning resulted in a temporary reduction in lymphocyte subsets, most notably of γδ lymphocytes. Considered together, abrupt weaning transiently impaired immune function in beef calves and thus, in terms of immunocompetence, weaning management strategies with the potential to minimise stress in beef calves should be considered.

## Methods

All animal procedures performed in this study were conducted under experimental licence from the Irish Department of Health and Children in accordance with the Cruelty to Animals Act 1876 and the European Communities (Amendment of Cruelty to Animals Act 1876) Regulation 2002 and 2005.

### Animal management and experimental design

Sixteen, spring-born (mean date of birth (s.d); 23 March (18.2) d) single-suckled, castrated, male calves of Limousin × Holstein-Friesian and Simmental × Holstein-Friesian dams and Simmental and Limousin sires, respectively, were used in this study. Cows and calves were rotationally grazed together on a predominantly perennial ryegrass-based sward from April until housing in a slatted floor shed at the end of the grazing season (13 November). On the day of housing (d 0), calves were moved to a handling yard adjacent to the paddock. Calves (mean age (s.d.) 235 (18.2) d; mean weight (s.d.) 310 (31.1) kg) were blocked by genotype, age, and weight and then randomly assigned within block to one of two treatments (i) abruptly weaned (W; n = 8) or (ii) non-weaned (control) (C; n = 8). Weaned calves were housed in pens without their dams (4 calves per pen), whereas non-weaned (control calves) were housed with their dams (2 cow-calf pairs per pen). Space allowance within the pens was equal for animals. Pens were equipped with automatic drinkers and animals were offered grass silage *ad libitum*. Cows that were separated from their calves were housed and had no auditory or visual contact with their calves.

### Rectal Body Temperature

Rectal body temperature of calves was recorded before blood sampling on d -7, 0 (housing), 2, 7, and 14 using a digital thermometer (Jørgen Kruuse; Marslev, Denmark).

### Blood Collection

Blood was collected into vacutainers (Vacuette, Cruinn Diagnostics, Ireland), containing the appropriate anticoagulant for subsequent haematological and flow cytometric analysis via direct jugular venipuncture using mild restraint in a holding chute at day (d) -7, 0 (housing), 2, 7 and 14. Blood samples were transported to the laboratory, stored at ambient temperature and processed within 3.5 h.

### Total and differential leukocyte populations

Total leukocyte, neutrophil, lymphocyte, monocyte, eosinophil, and basophil were determined from K_3_EDTA anti-coagulated blood (6 mL) using an automated haematology analyzer (ADVIA 2120, Bayer Healthcare, Siemens, UK) equipped with software for bovine blood.

### Leukocyte immunostaining

Acid citrate dextrose anti-coagulated blood (8 mL) was used to for leukocyte immunostaining using a whole blood assay [57]. Briefly, duplicate 1 mL aliquots of whole blood were transferred to a 5 mL test tube (Sarstedt, Nümbreacht, Germany) and tubes were centrifuged at 250 × *g *for 5 mins at 4°C. After aspiration of supernatants, 3 mL of BD FACS lysing solution (BD Biosciences, Oxford, UK) was added for 10 mins at room temperature to lyse erythrocytes. Remaining leukocytes were resuspended in 1.5 mL of sheath fluid (Coulter Isoton II Diluent (Beckman Coulter UK Ltd., High Wycombe, UK) and counted using a Z1 Coulter Particle Counter (LABPLAN Ltd., Galway, Ireland). One hundred microliter aliquots of cell suspension (1 × 10^6 ^cells) were seeded into series of wells on a 96-well microtiter plate. Leukocyte immunostaining was carried out using a 2-antibody system, as described by [41], with the exception of the secondary (detection) antibody where FITC-labelled goat anti-mouse IgG F(ab')2 (Southern Biotech, Birmingham, AL, USA) was used following a 1/100 dilution with PBS-0.01 (w/v) % BSA. Sources, specificities, isotypes and working solutions of monoclonal and secondary antibodies are described in Table 4. All antibodies were diluted to final working concentration using PBS-0.01% (w/v) BSA (pH 7.2). Following this procedure, cells were fixed with 200 μl of 1% (v/v) paraformaldehyde, and further diluted with 800 μl of sheath fluid for immediate acquisition on a Partec CyFlow SL flow cytometer (Partec, Münster, Germany). Cells were kept at 4°C in the dark prior to acquisition.

**Table 4:** Antibodies used in the immunostaining of leukocyte surface markers in beef calves

Specificity	Cell types	Clone	Isotype	Working solution	Source^1^
CD4	T-helper/inducer cells	CC8	IgG_2_a	7 μL/mL	Serotec
CD8	T-cytotoxic/suppressor cells	CC63	IgG_2_a	7 μL/mL	Serotec
WC1	Subset of γδ T cells	CC15	IgG_2_a	7 μL/mL	Serotec
MHC class II	Antigen presenting cells (B cells, activated T cells)	H42A	IgG_2_a	7 μL/mL	VMRD
CD62L	L-selectin	BAQ92A	IgG_1_	14 μL/mL	VMRD
G1	Neutrophils (and eosinophils)	MM20A	IgG_1_	14 μL/mL	VMRD
CD45	All leukocytes (pan marker)^2^	CC1	IgG_1_	3.5 μL/mL	Serotec

^1^Serotec (Oxford, UK), VMRD (Pullman, WA, USA).

^2^Used to differentiate leukocyte populations and exclude debris (gate analysis).

### Phagocytic and oxidative burst activity assays

The phagocytic and oxidative burst activity of neutrophils was determined in lithium heparin anti-coagulated blood (8 mL) using the Phagotest kit and Bursttest (PhagoBurst) kit (Orpegen Pharma, Heidelberg, Germany) following the manufacturer's instructions, with modifications [58], on a Partec CyFlow SL flow cytometer (Partec Gmbh, Münster, Germany). Duplicate tests for each sample were performed.

### Flow cytometric analysis

*Immunophenotypes*. A minimum of 30,000 events were acquired and analyzed using FloMax software (Partec GmbH, Münster, Germany). Lymphocytes and neutrophils were gated from all other leukocyte populations based on their forward and side scatter characteristics on dot plots and were confirmed using CD45 (pan leukocyte) staining [59]. The percentage lymphocytes staining positive for CD4, CD8, WC1, and MHC class II, and percentage of neutrophils staining positive for G1 was recorded. Surface expression of CD62L was recorded as mean fluorescence intensity (MFI) of CD62L-staining positive neutrophils. A threshold for positive staining cells was set using histograms identifying irrelevant isotype controls and PBS-0.01 (w/v) % BSA only treated leukocytes.

*Phagocytic and oxidative burst activity assays*. Data were acquired from 15,000 cells per sample and analysis was carried out using FloMax software (Partec Gmbh, Münster, Germany). For each sample, the percentage phagocytosis positive and oxidative burst positive cells within the neutrophil gate were recorded.

### Acute phase protein response (fibrinogen and haptoglobin)

Blood collected into vacutainer tubes containing lithium heparin (8 mL) and sodium citrate (4.5 mL) was used to determine the concentration of haptoglobin and fibrinogen, respectively. Plasma was harvested following centrifugation at 1600 × *g *at 4°C for 15 min and stored at -80°C until assayed. Concentration of plasma haptoglobin was measured using an automatic analyzer (spACE, Alfa Wassermann, Inc., West Caldwell, NJ, USA) and commercial assay kit (Tridelta Development Ltd., Wicklow, Ireland) according to the manufacturer's procedure as described by [60]. Concentration of fibrinogen was measured using an automatic analyzer (spACE, Alfa Wassermann, Inc., West Caldwell, NJ, USA) using a previously described method [61].

### Statistical Analysis

All statistical analysis was performed using SAS/STAT for Windows [62]. Data were tested for normality using PROC UNIVARIATE, and data (neutrophil G1 data only) that did not meet parametric assumptions were transformed by a square root transformation prior to statistical analysis. Data were then analyzed as repeated measures using the PROC MIXED procedure of SAS with an unstructured covariance matrix within animal. The effects of treatment, sampling time, genotype and all possible interactions were listed in the model statement. Genotype was not significant (*P *> 0.05) and was not included in the model. Day -7 was included as a covariate but this was not significant (*P *> 0.05) and was omitted from the model. Baseline is defined as day 0 for each variable in the description of the results and representation of the data. Least squares means were estimated, differences between least squares means were determined using the Tukey-Kramer test for multiple comparisons, and the associated *P*-values presented were derived from the statistical analysis of the data using the model described above. A probability of *P <*0.05 was selected as the level of significance.

## Authors' contributions

BE and MMcG designed the study. EML and BE performed the experiments. BE and EML analysed the data and EL prepared the manuscript. EML, BE, MMcG and SD contributed to, read and approved the final manuscript.
